# Supplementary material for: MetaRibo-Seq measures translation in microbiomes
Source: Nat Commun. 2020 Jun 29;11:3268. doi: 10.1038/s41467-020-17081-z (PMC7324362; doi:10.1038/s41467-020-17081-z)
Supplement: Supplementary file 10 — Supplementary Data 7 [file 41467_2020_17081_MOESM10_ESM.zip › File2/Confidence_High_Taxonomy/30218_out.krona.html]

Javascript must be enabled to view this page.

members
magnitude
magnitudeUnassigned
count
unassigned
taxon
rank

30218\_out

128

128
2
superkingdom

128
phylum
976

class
200643
128

171549
order
128

128
815
family

128
genus
816


SRS020622\_contig\_number\_2064
species
674529
1

1
species
1263054

SRS053649\_contig\_number\_9903

species
291644
1

SRS075773\_contig\_number\_4129


SRS011134\_contig\_number\_18965SRS013215\_contig\_number\_855SRS013940\_contig\_number\_18991SRS014459\_contig\_number\_8393SRS014613\_contig\_number\_11378SRS014855\_contig\_number\_5420SRS015431\_contig\_number\_55113SRS015694\_contig\_number\_3747SRS015854\_contig\_number\_642SRS016018\_contig\_number\_746SRS016095\_contig\_number\_3458SRS016267\_contig\_number\_13SRS016753\_contig\_number\_262SRS016840\_contig\_number\_1677SRS016897\_contig\_number\_530SRS016954\_contig\_number\_3923SRS018351\_contig\_number\_9211SRS018817\_contig\_number\_9335SRS019286\_contig\_number\_3553SRS019381\_contig\_number\_8SRS019397\_contig\_number\_2939SRS019787\_contig\_number\_705SRS019968\_contig\_number\_8775SRS020233\_contig\_number\_27366SRS021153\_contig\_number\_278SRS021219\_contig\_number\_265SRS022137\_contig\_number\_20224SRS022609\_contig\_number\_29561SRS022713\_contig\_number\_707SRS023346\_contig\_number\_540SRS023526\_contig\_number\_10642SRS024075\_contig\_number\_200SRS024549\_contig\_number\_908SRS024625\_contig\_number\_7492SRS024663\_contig\_number\_12329SRS042966\_contig\_number\_12491SRS043001\_contig\_number\_108SRS043667\_contig\_number\_82SRS045713\_contig\_number\_4953SRS046502\_contig\_number\_9513SRS046717\_contig\_number\_4437SRS048870\_contig\_number\_10380SRS049773\_contig\_number\_5532SRS049896\_contig\_number\_2392SRS049959\_contig\_number\_29385SRS053356\_contig\_number\_43098SRS054905\_contig\_number\_1752SRS055982\_contig\_number\_869SRS056273\_contig\_number\_454SRS056519\_contig\_number\_10124SRS056537\_contig\_number\_34327SRS057478\_contig\_number\_760SRS057717\_contig\_number\_891SRS062654\_contig\_number\_1211SRS063489\_contig\_number\_313SRS063813\_contig\_number\_2648SRS063928\_contig\_number\_294SRS064276\_contig\_number\_28998SRS065504\_contig\_number\_10337SRS074670\_contig\_number\_271SRS076804\_contig\_number\_14530SRS077127\_contig\_number\_379SRS077194\_contig\_number\_12196SRS077641\_contig\_number\_13632SRS078242\_contig\_number\_9762SRS098061\_contig\_number\_9445SRS098073\_contig\_number\_11269SRS098881\_contig\_number\_7343SRS101433\_contig\_number\_4814SRS1041090\_contig\_number\_6706SRS1041116\_contig\_number\_794SRS1041118\_contig\_number\_2201SRS1041129\_contig\_number\_10446SRS1041130\_contig\_number\_248SRS1041132\_contig\_number\_6616SRS1041139\_contig\_number\_163SRS1041145\_contig\_number\_11755SRS104311\_contig\_number\_28079SRS104636\_contig\_number\_27710SRS104693\_contig\_number\_3788SRS104975\_contig\_number\_13174SRS105082\_contig\_number\_18979SRS105153\_contig\_number\_1881SRS1055022\_contig\_number\_2050SRS1055043\_contig\_number\_18894SRS1055067\_contig\_number\_8587SRS1055069\_contig\_number\_1303SRS1055095\_contig\_number\_241SRS1055099\_contig\_number\_3295SRS140513\_contig\_number\_8464SRS142503\_contig\_number\_5769SRS142923\_contig\_number\_1109SRS143085\_contig\_number\_2309SRS143148\_contig\_number\_14972SRS143372\_contig\_number\_2913SRS143598\_contig\_number\_34SRS143876\_contig\_number\_23069SRS144537\_contig\_number\_35099SRS145308\_contig\_number\_1126SRS146813\_contig\_number\_6873SRS147139\_contig\_number\_31786SRS147977\_contig\_number\_1710SRS148970\_contig\_number\_1717SRS149075\_contig\_number\_1178SRS149325\_contig\_number\_15926SRS893170\_contig\_number\_8SRS893172\_contig\_number\_9477SRS893230\_contig\_number\_3961SRS893253\_contig\_number\_456SRS893256\_contig\_number\_40SRS893288\_contig\_number\_854SRS893378\_contig\_number\_1687SRS893382\_contig\_number\_2430SRS893383\_contig\_number\_13523
species
818
114

2
species
93974

SRS013965\_contig\_number\_41345SRS063370\_contig\_number\_2422

1
species
2292942

SRS144714\_contig\_number\_contig-100\_443.134820

3
species
46506

SRS097920\_contig\_number\_9792SRS101376\_contig\_number\_3546SRS104327\_contig\_number\_59


SRS015782\_contig\_number\_13380SRS017103\_contig\_number\_488
2
47678
species

246787
species
1

SRS148784\_contig\_number\_922

2
821
species

SRS011405\_contig\_number\_777SRS148253\_contig\_number\_9913
